# Sexual Dimorphism in the Age of Genomics: How, When, Where

**DOI:** 10.3389/fcell.2019.00186

**Published:** 2019-09-06

**Authors:** Daniel F. Deegan, Nora Engel

**Affiliations:** Fels Institute for Cancer Research, Lewis Katz School of Medicine, Temple University, Philadelphia, PA, United States

**Keywords:** sexual dimorphism, sex-biased expression, transcriptional regulation, epigenetics, embryogenesis

## Abstract

In mammals, sex chromosomes start to program autosomal gene expression and epigenetic patterns very soon after fertilization. Yet whether the resulting sex differences are perpetuated throughout development and how they connect to the sex-specific expression patterns in adult tissues is not known. There is a dearth of information on the timing and continuity of sex biases during development. It is also unclear whether sex-specific selection operates during embryogenesis. On the other hand, there is mounting evidence that all adult tissues exhibit sex-specific expression patterns, some of which are independent of hormonal influence and due to intrinsic regulatory effects of the sex chromosome constitution. There are many diseases with origins during embryogenesis that also exhibit sex biases. Epigenetics has provided us with viable mechanisms to explain how the genome stores the memory of developmental events. We propose that some of these marks can be traced back to the sex chromosomes, which interact with the autosomes and establish sex-specific epigenetic features soon after fertilization. Sex-biased epigenetic marks that linger after reprograming may reveal themselves at the transcriptional level at later developmental stages and possibly, throughout the lifespan. Detailed molecular information on the ontogeny of sex biases would also elucidate the sex-specific selective pressures operating on embryos and how compensatory mechanisms evolved to resolve sexual conflict.

## Introduction

In the age of genomics, it has become ever more obvious that the long-known differences between males and females in health, longevity, disease risk and presentation, and response to therapy have genetic and epigenetic foundations (Yang et al., 2006; Isensee et al., 2008; Singmann et al., 2015; Chen et al., 2016; Mayne et al., 2016; Gershoni and Pietrokovski, 2017; McCormick et al., 2017). It is now clear that every adult somatic cell, to a greater or lesser degree, exhibits sex biases in gene expression and epigenetic profile in human and non-human primates, rodents, and bovines (Yang et al., 2006; Blekhman et al., 2010). There is also a growing realization that sex differences are the result of complex interactions between the sex hormones, genetic variability, and the environment, all of which operate on the background of the intrinsic effects of the sex chromosome composition, i.e., XX for females and XY for males (Arnold, 2014; Engel, 2018; Raznahan et al., 2018; Khramtsova et al., 2019).

Although the driver of sex differences in mammals has traditionally been considered to be the so-called sex determination pathway, sex-specific transcriptional and epigenomic profiles are present in the embryo very soon after fertilization in a range of mammals, i.e., well before the development of the gonads (Burgoyne et al., 1995; Bermejo-Alvarez et al., 2010; Lowe et al., 2015; Hansen et al., 2016). Moreover, male and female embryos exhibit different susceptibilities to environmental factors during early gestation (Jenkins et al., 2007; Gabory et al., 2012; Bale, 2016; Bansal et al., 2017). These differences reflect the early sexual identity of the embryo (Hansen et al., 2016) and of the placenta (Nugent et al., 2018), contrasting sharply with the prevailing view of sex-neutral development prior to gonadogenesis (Dewing et al., 2003). Long before sex hormones appear, the primary sex-determining factor is the imbalance in sex chromosome composition (Blecher and Erickson, 2007; Arnold, 2012).

If sex differences at the molecular level begin at such an early stage, the question is, do those differences matter and what are their contributions to sex differences that become apparent later in life? Do these sex biases matter more for some tissues than for others? Do expression and epigenetic sex biases wax and wane over the course of embryogenesis? How does the sex-specific molecular skewing inform on compensatory mechanisms that operate on males and females during embryogenesis from an evolutionary standpoint? The goal of this article is to identify gaps in our knowledge that impede us from answering these questions.

## Sex Biases in Pre-Implantation Embryos

### The Sex-Specific Regulatory Environment in Early Embryogenesis

Two observations justify how sex-biased expression in pre-implantation embryos could establish male and female-specific transcriptional and epigenetic legacies that become apparent at later stages in development. First, a number of dosage-dependent regulatory factors are expressed in a sex-biased manner in pre-implantation embryos, some of which are encoded on the X and Y chromosomes. Many transcription factors (TFs) and epigenetic regulators must be expressed at the appropriate levels for proper activation or repression of their downstream target genes. In fact, TFs are overrepresented in haploinsufficiency disorders (McKusick, 2002; Seidman and Seidman, 2002)^1^, in which mutations inactivating one allele produce a reduction by half in the protein levels of the TF. Second, there are precedents for epigenetic marks present after fertilization to persist after implantation. For example, genomic imprints from each parental genome are maintained throughout the genome-wide pre- and post-implantation reprograming processes (Barlow and Bartolomei, 2014; Engel, 2015).

In principle, variations in TF levels can change the response of their target genes or alter their affinity to their cognate sites (Chen et al., 2014), because in contrast to prokaryotes, transcriptional activation in eukaryotes is not always an all-or-none response. Promoters and enhancers are more or less sensitive to TF concentrations depending on the number of binding sites for specific TFs (Badis et al., 2009; Lambert et al., 2018). In addition, TFs often act synergistically or in multimers, so higher or lower levels can result in changes in downstream effects (Jolma et al., 2015).

Dosage also affects epigenetic factors (EFs), such as DNA-methyltransferases and histone modification enzymes. Because these usually act in large complexes, changes in the levels of protein components of these complexes can alter their stoichiometry and function (Veitia and Birchler, 2015; Ori et al., 2016). In turn, some TFs are sensitive to the chromatin environment (Blattler and Farnham, 2013; Inukai et al., 2017; Yin et al., 2017), so male- and female-specific epigenetic differences in binding sites can change their availability.

Unfortunately, there is a dearth of experimental data to determine how dosage differences in TFs and EFs shift transcriptomes, much less phenotypes, in mammalian model systems. ChIP data showing that sex-biased TFs are distributed differentially across the genome or that they activate their targets differentially would go a long way toward understanding dosage effects of regulatory factors. Making ChIP more quantitative and more sensitive, and expanding the availability of ChIP-grade antibodies for regulatory factors is a pre-requisite. Perhaps technology that allows us to tag TFs by genetic engineering will solve some of these issues (Savic et al., 2015). The ability to finely tune the levels of TFs is also necessary to determine if subtle variations have downstream consequences.

### X Chromosome Inactivation (XCI) in Female Embryos

One of the best-studied events distinguishing male and female pre-implantation embryos is that females undergo X chromosome inactivation (XCI). XCI in placental mammals is a dosage compensation mechanism that transcriptionally silences the majority of genes on one of the X chromosomes in females. Because males have a single X chromosome, this ensures dosage equivalence between males and females. The long non-coding RNA *Xist* becomes highly expressed on one X chromosome, coating the entire chromosome and triggering the accumulation of DNA methylation and condensing histone modifications, ultimately resulting in heterochromatinization (Disteche and Berletch, 2015; Sahakyan et al., 2018).

Two consequences result from this massive epigenetic overhaul of an entire chromosome. First, female embryos are developmentally delayed relative to male embryos until XCI is complete (Thornhill and Burgoyne, 1993; Schulz et al., 2014). It is well-established that XCI is intimately tied to cell differentiation, at least in the mouse (Lessing et al., 2013; Payer and Lee, 2014), and its failure is lethal (Takagi and Abe, 1990; Marahrens et al., 1997). Thus, in addition to the effects of sex-biased expression of TFs, the delay in XX embryos opens a window of opportunity for TFs and EFs to act on the female genome in a sex-specific manner, even if they are not expressed in a sex-biased manner. On the other hand, the male genome may undergo specific modifications as a consequence of not needing to inactivate an X chromosome.

Another consequence of XCI that has been hypothesized is that the inactive X is a sink for epigenetic factors, altering their relative concentrations between males and female, with possible consequences for autosomal regulation (Wijchers et al., 2010; Arnold et al., 2012). The decrease in availability of EFs in females would introduce differences in the chromatin status of regulatory sequences. In turn, this would introduce a variation in how the genome is read and regulated in the female embryo. Both of these scenarios require experimental validation with sensitive genomic and proteomic tools that allow interrogation of single sexed embryos before and after XCI to determine whether females are on a different developmental clock and whether specific epigenetic factors are indeed substantially diminished relative to male embryos.

The process of XCI is stochastic in the embryo and the choice of the X chromosome to be inactivated is heritable. This means that female placental mammals are mosaics because in some cells the paternally inherited X chromosome is inactive whereas in other cells it is the maternally inherited X which is inactive (Migeon, 2007). As a result, expression of X-linked allelic variants will vary in different cell lineages (Wu et al., 2014). If the alleles exhibit variation in their expression levels, female cells in which the maternal X is active can have expression levels of X-linked genes that differ from those in male cells.

Although the majority of genes are silenced on the inactive X chromosome, a number of genes escape XCI and remain more highly expressed in female cells after implantation, contributing to sex biases in gene expression throughout the lifespan of the organism (Disteche and Berletch, 2015; Balaton and Brown, 2016).

## Post-Implantation Embryogenesis and Beyond

Implantation signals a major reprograming of the genome, concomitant with lineage determination. If sex-biased epigenetic landscapes can weather the *de novo* DNA methylation and chromatin re-structuring that ensues, it remains to be determined which specific epigenetic marks identify the cell as male or female. If, on the other hand, implantation erases all sex biases between XX and XY embryos, there are still genes encoded on the sex chromosomes that are differentially expressed before the appearance of sex hormones that could lead to sex-biased autosomal gene expression. Such is the case of Y-linked genes, absent in female cells, X-linked allelic variants and genes that escape XCI altogether (Disteche and Berletch, 2015). It has been reported that XCI “escapees” present some degree of tissue-specificity in adult tissues (Berletch et al., 2015; Balaton and Brown, 2016; Tukiainen et al., 2017). Therefore, we need a detailed, lineage-specific catalog of what genes escape XCI over the course of development and how they affect transcriptional outcomes.

Studies in multiple non-mammalian models have revealed sex-biased expression of many genes not necessarily related to sexual function throughout embryogenesis (Mank et al., 2010; Ma et al., 2018). Such detailed characterization of the fluctuations in transcriptional and epigenetic sex biases during development is lacking for mammals. Therefore, tissue-specific developmental time-series data are needed to begin to answer these questions experimentally.

## Are Sex Differences During Embryogenesis Meaningful From an Evolutionary Standpoint?

Evolutionary conflict arises between the sexes when their fitness interests diverge. Because males and females share most of their genomes, genes common to both sexes encode many of their shared traits (Cox and Calsbeek, 2009; Hosken et al., 2019). Sexually antagonistic selection emerges when optimal fitness for traits differs, leading to intra-locus sexual conflict. For example, intra-locus conflict arises when expression of a gene is beneficial in one sex but detrimental in the other. Contradictory selection pressures can lead to sub-optimal expression levels for each sex, with subsequent regulatory mechanisms evolving to offset the less-than-optimal expression level. Thus, sex-biased gene expression can be indicative of ongoing or resolved intra-locus sexual conflict (Parsch and Ellegren, 2013; Rowe et al., 2018).

Forces generating expression differences are expected to be maximal in the adults, because this is when reproductive interests diverge. However, if we envision sexual differentiation as a progressive developmental process, with independent and combined contributions from the sex chromosomes and sex hormones, it is possible that expression patterns of early embryos are also under sex-specific selection pressures and that sex-biased expression during development indicates sexual antagonism (Ingleby et al., 2015). Because we lack detailed sex-stratified data across the whole life cycle in mammals, we do not know how sex-biased transcription contributes to the male and female phenotypes, much less all of the genes involved. For example, some sex biases may need to be expressed continuously throughout development, while others may be transient, setting the stage for later sexual dimorphism.

A different mechanism of uncoupling the genetic architecture between males and females involves gene duplication and the evolution of sex-specific regulatory mechanisms for each duplicate (Wyman et al., 2012). Especially in the case of mammals, with their greatly expanded families of TFs, it would be interesting to investigate if different paralogs enable conflict resolution by harboring divergent regulatory sequences that direct sex-specific expression.

Although the majority of genes that contribute to sexually dimorphic traits are autosomal and shared between the sexes, the sex chromosomes are a separate solution to sexual conflict, expanding the range of sexual differences at the level of expression that can exist (Hosken et al., 2019; Wright et al., 2019). The special nature of the sex chromosomes is related to evolutionary forces that have driven their differentiation and the compensatory mechanisms that allow male cells to tolerate the presence of a single X chromosome (Skaletsky et al., 2003; Graves, 2016; Arnold, 2019). These forces are independent, but can interact with those related to the divergent niches of males and females in reproduction. Offsetting the imbalance in the sex chromosomes is necessary either because genes on the sex chromosomes participate in complex regulatory networks or because they encode components of dosage-sensitive protein complexes (Bellott et al., 2014; Veitia and Potier, 2015). Compensation is partially achieved by XCI in female cells. However, very little is known about the adjustments of the autosomes to the imbalance of sex chromosomes in males and females, which in principle could give rise to sex-biased expression of autosomal genes at any point in development (Veitia et al., 2015).

Detailed molecular information across all stages of development for males and females would allow us to test the major hypotheses on the ontogeny and evolutionary significance of sex biases by integrating functional studies of individual genes with systems-level analyses and identifying similarities and differences across a range of species.

## Systems Biology of Sex Biases

Some genes are expressed with less than a twofold difference between the sexes (Arnold et al., 2009; Werner et al., 2017). These differences may be considered trivial, but the systems biology revolution has highlighted that genes are interconnected in complex networks and that small differences in multiple genes can shift transcriptional and phenotypic outcomes. Considering that some sexually dimorphic traits are extremely complex, many small-effect loci are likely to underlie these traits. Expression variation quantitative trait locus (eQTL) mapping of sex-biased expression in mice support this expectation (Yang et al., 2006; van Nas et al., 2010).

A recent mandate from the NIH to include sex as a biological variable in all studies has adrenalized the interest in sex differences in disease risk and susceptibility (Clayton and Collins, 2014). Significant inroads have been made in characterizing sex biases in gene expression and epigenetic features in a variety of adult tissues. Modeling of regulatory networks in adult human and mouse tissues have shown surprising differences in regulatory architecture between males and females (Chen et al., 2016; Gershoni and Pietrokovski, 2017; Karp et al., 2017; Shen et al., 2017).

**FIGURE 1:**
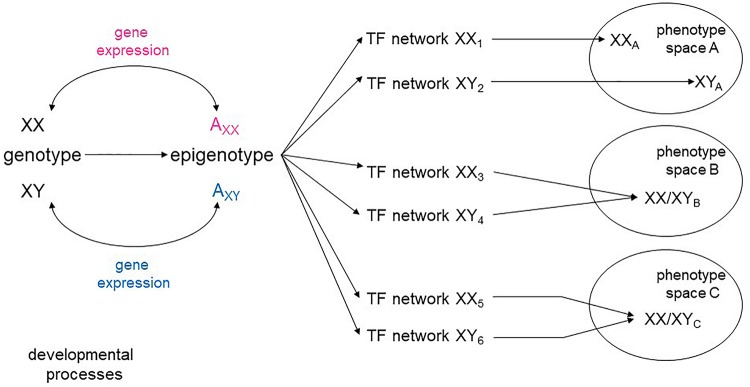
Sex-specific transcriptional networks and phenotype maps. Schematic representation of the relationships between genotype, transcriptional networks, and final phenotype during development. Male and female genotypes, represented as XY and XX produce distinct epigenotypes, with effects on and counter-effects from the autosomes (A). Modifications of the epigenotype on the autosomes lead to transcriptional changes that in turn influence expression from the sex chromosomes. Different transcription factor (TF) networks can either determine distinct phenotypes (space A) or converge to an equivalent phenotype (spaces B, C).

The range of continuously evolving analytical tools opens the possibility of looking at the aggregate pattern of sex-biased expression to reveal sex-specific modules within the global networks that specify cellular types. There is a high degree of plasticity in developmental pathways, with a variety of intermediate states leading to the same phenotypic space (Briggs et al., 2017; Figure 1). It is conceivable, then, that sex skews some parts of a network encoding a cellular phenotype, while not affecting others. It is also possible that some cell types may require a greater degree of molecular convergence between the sexes than others. Sex-stratified transcriptional and epigenetic data from embryos would also allow a more complete understanding of how the appearance of sex hormones affect developmental processes beyond the reproductive system.

## Speculations and Future Directions

**FIGURE 2:**
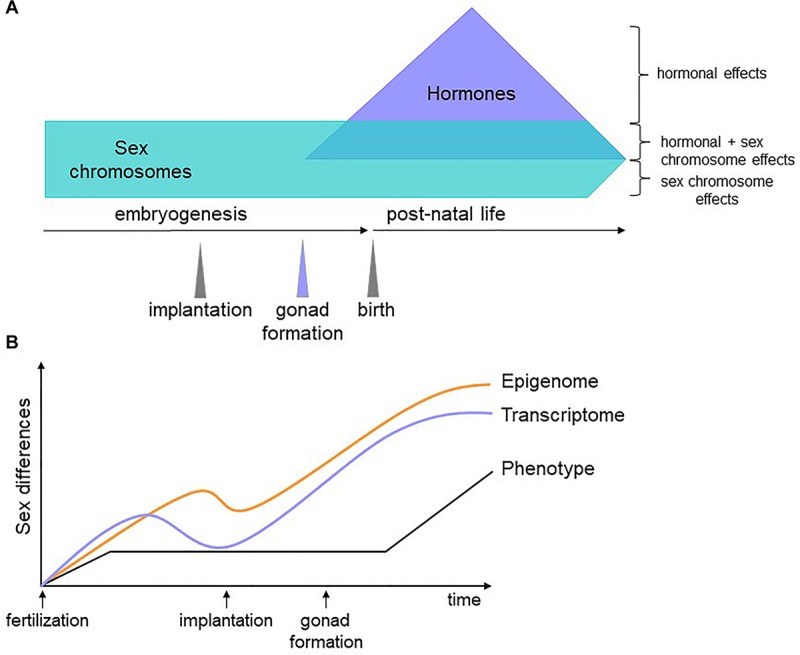
Schematic of our hypothesis. **(A)** Sex biases have different origins depending on the developmental stage of the organism. Before gonadogenesis, sex chromosomes are the primary determinants of sex differences. Sex hormones influence the transcriptome and epigenome independently of and in combination with sex chromosome effects. **(B)** Soon after fertilization, male and female cells have sex-specific transcriptomes, epigenomes, and phenotypes (for example, male embryos grow faster than female embryos). At implantation, lineage determination begins and gene expression differences are reduced. Epigenetic marks, however, are less constrained and some are maintained, affecting gene expression, and phenotype later in development. Once specific lineages are established, differences in gene expression increase again due to environmental, hormonal and genetic factors, some of which act on sex-specific epigenetic features established prior to differentiation.

We propose that genes encoded on the sex chromosomes act on autosomal genes to generate a differential regulatory and epigenetic landscape upon which later factors, such as hormones, act to counter or compound sex biases. Because the epigenome does not necessarily affect transcription until stage-specific TFs appear, epigenetic sex biases established in early development could persist and contribute to sex-specific phenotypes at later time points (Figure 2). Testing this hypothesis will first require identifying the nature of sex-biased epigenetic marks, with DNA methylation an obvious candidate. Then, we must gather and integrate dynamic, sex-stratified epigenetic, expression, and proteomic data throughout embryogenesis. The degree to which molecular sex differences are compensated for between the sexes are likely to be tissue-specific, with some cell types requiring greater molecular convergence than others for proper functionality. This can be revealed with detailed tissue-specific analyses, a time-consuming but certainly worthwhile effort. The role of sex hormones in these processes can then be inferred and validated with *in vivo* manipulations in animal models. This will pave the way for connecting sex biases during development to adult phenotypes.

## Concluding Remarks

There are many outstanding questions on the significance and extent of sex biases in gene expression and the epigenome, especially during mammalian embryogenesis. We have evidence that the sex chromosomes and autosomes are engaged in a regulatory dialogue very soon after fertilization, but the implications for the specification of cell types and organogenesis are unclear. Stratification by sex of existing and forthcoming data, in combination with experimental validation, will allow us to determine whether early sex biases have ramifications across the lifespan. Translation of these insights into humans will require incorporating studies on how genetic background and environmental factors influence sex differences. These studies have practical value in understanding how sex functions as a variable in the developmental origins of disease (Barker, 2004). If in fact there are sex-specific evolutionary pressures acting on embryos, finding the specific genes involved and the networks they are embedded in will inform us on the compensatory mechanisms that allow males and females to develop into healthy organisms. The coming years will undoubtedly be exciting times in the field of sex differences.

## Author Contributions

DD contributed to the bibliography compilation and review of the manuscript content. NE wrote and reviewed the manuscript. Both authors approved the final version of the manuscript.

## Conflict of Interest Statement

The authors declare that the research was conducted in the absence of any commercial or financial relationships that could be construed as a potential conflict of interest.

## Footnotes

1 https://www.ncbi.nlm.nih.gov/omim
